# Comparison of Osteogenic Potentials of Dental Pulp and Bone Marrow Mesenchymal Stem Cells Using the New Cell Transplantation Platform, CellSaic, in a Rat Congenital Cleft-Jaw Model

**DOI:** 10.3390/ijms22179478

**Published:** 2021-08-31

**Authors:** Jinzhao Lyu, Yoshiya Hashimoto, Yoshitomo Honda, Naoyuki Matsumoto

**Affiliations:** 1 Department of Orthodontics, Osaka Dental University, 8-1 Kuzuhahanazonocho, Hirakata, Osaka 573-1121, Japan; lyu-j@cc.osaka-dent.ac.jp (J.L.); matsumoto-n@cc.osaka-dent.ac.jp (N.M.); 2 Department of Biomaterials, Osaka Dental University, 8-1 Kuzuhahanazonocho, Hirakata, Osaka 573-1121, Japan; 3 Department of Oral Anatomy, Osaka Dental University, 8-1 Kuzuhahanazonocho, Hirakata, Osaka 573-1121, Japan; honda-y@cc.osaka-dent.ac.jp

**Keywords:** stem cells, Cellnest™, CellSaic, rDPSCs, rBMSCs, regeneration, osteogenic differentiation, bone repair, rat congenital cleft-jaw model

## Abstract

Scaffolds stimulate cell proliferation and differentiation and play major roles in providing growth and nutrition factors in the repair of bone defects. We used the recombinant peptide Cellnest™ to prepare the three-dimensional stem cell complex, CellSaic, and evaluated whether CellSaic containing rat dental pulp stem cells (rDPSCs) was better than that containing rat bone marrow stem cells (rBMSCs). rDPSC-CellSaic or rBMSC-CellSaic, cultured with or without osteogenic induction medium, formed the experimental and control groups, respectively. Osteoblast differentiation was evaluated in vitro and transplanted into a rat model with a congenital jaw fracture. Specimens were collected and evaluated by microradiology and histological analysis. In the experimental group, the amount of calcium deposits, expression levels of bone-related genes (RUNX2, ALP, BSP, and COL1), and volume of mineralized tissue, were significantly higher than those in the control group (*p* < 0.05). Both differentiated and undifferentiated rDPSC-CellSaic and only the differentiated rBMSC-CellSaic could induce the formation of new bone tissue. Overall, rBMSC-CellSaic and rDPSC-CellSaic made with Cellnest™ as a scaffold, provide excellent support for promoting bone regeneration in rat mandibular congenital defects. Additionally, rDPSC-CellSaic seems a better source for craniofacial bone defect repair than rBMSC-CellSaic, suggesting the possibility of using DPSCs in bone tissue regenerative therapy.

## 1. Introduction

In orthodontic treatment, insufficient bone mass, such as bone defects or bone resorption [1,2], causes problems in the treatment. Although autologous bone transplantation is the gold standard for bone regeneration, the source of bone mass limits its practical application in clinical practice [3,4]. Regenerative medicine provides revolutionary methods for such defects, including the use of scaffolds, stem cells, and cell signaling molecules to promote defect repair [5].

In previous tissue engineering research, most of the cells grown on two-dimensional (2D) substrates showed simplified morphological and character changes, which caused conditions to be quite different from the natural microenvironment [6]. Two-dimensional culture methods cannot replicate cell–cell and cell–extracellular matrix (ECM) interactions present in tissues [7]. To overcome the limitations of the 2D culture system, Li et al. developed various three-dimensional (3D) culture methods, such as the hanging drop method, spontaneous sphere formation, and high-porosity bionic scaffolds to simulate the tissue microenvironment and improve differentiation efficiency [8]. According to previous reports, 3D culture may better reflect the in vivo microenvironment [9].

The 3D culture that attracted our attention utilizes a new type of scaffold. Fujifilm has developed a new type of 3D-absorbable biomaterial based on the α-1 sequence of human type I collagen termed “Cellnest^TM^”. We fused Cellnest^TM^ with multipotent stem cells to form a three-dimensional structure termed “CellSaic”. “CellSaic” is a term coined from “Cell” and “Mosaic” [10]. The CellSaic size is approximately 50–100 µm [11]. The complex petal-like shape of the CellSaic is the most significant difference from other 3D culture systems, which increases the surface area for cell adhesion and ensures that gaps in cell aggregates allow nutrients and waste to pass through [12,13]. Moreover, unlike traditional animal collagen, the recombinant peptide (RCP) was produced by the yeast *Pichia pastoris* and carries no risk of infection, such as bovine spongiform encephalopathy [11,14]. It not only supports cell survival, but also provides optimal conditions for the synthesis of substrates [15].

At the cellular level, mesenchymal stem cells (MSCs) are multipotent stem cells that self-renew and differentiate into multiple lineages [16,17,18,19]. Bone marrow mesenchymal stem cells (BMSCs) have a high osteogenic ability. They are considered one of the most suitable multipotent stem cells for bone regeneration; therefore, they are widely used for comparisons of efficiency with other cell sources [20,21,22,23]. However, BMSCs have some practical shortcomings in clinical applications, including painful collection methods, damage to the donor site, and a reduced proliferation and differentiation ability as the donor ages [24].

Dental pulp stem cells (DPSCs) extracted from the pulp of human molars are also considered a high-quality source of MSCs in tissue engineering [25]. They have several advantages in bone regeneration, including a high proliferation rate, excellent osteogenic differentiation potential, and good paracrine and immunomodulatory properties [26,27]. In addition, it is simple to isolate DPSCs from easily accessible extracted and discarded teeth, thus providing a rich source of cells for regenerative medicine, with minimal risk of complications [28,29]. The wide potential of DPSCs for applications in regenerative pulp therapy has aroused great interest [30]. Both BMSCs and DPSCs are promising sources for bone regeneration. Some studies have reported that DPSCs exhibit better performance than BMSCs [31,32], while other studies have shown no significant differences between them [33].

In addition, when stem cells are implanted into different anatomical areas or animal models, their osteogenic effects may show different regeneration potentials [34]. At present, the regeneration efficiency of BMSCs and DPSCs in a mandibular congenital defect model is unclear. The present study evaluated the possibility of culturing rat BMSCs and DPSCs using the CellSaic platform (hereinafter referred to as rBMSC-CellSaic or rDPSC-CellSaic) in osteogenic differentiation and nondifferentiation media. This study also investigated whether they can be used in the rat mandibular congenital defect model to induce bone formation. The findings of this study may be of practical value and provide prospective research ideas for future clinical applications. To the best of our knowledge, this study is the first to use CellSaic to evaluate the osteogenicity of rBMSCs and rDPSCs in a rat congenital cleft-jaw model.

## 2. Results

### 2.1. Characterization and Surface Antigen Analysis of rBMSCs and rDPSCs

**Figure 1:**
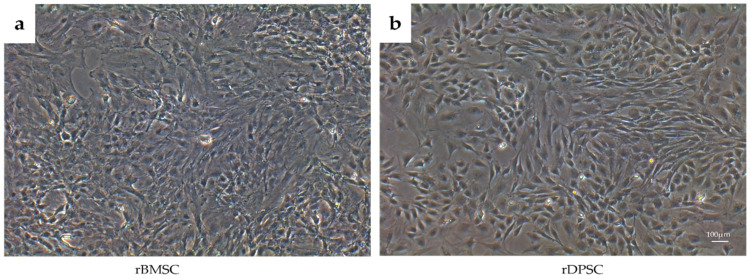
Morphology and proliferation of rBMSCs and rDPSCs. Cells showed a spindle-like fibrous morphology of uniform shape and size. (**a**) rBMSC cells after 3 days of culture; (**b**) rDPSC cells after 3 days of culture (Scale bar = 100 µm).

**Figure 2:**
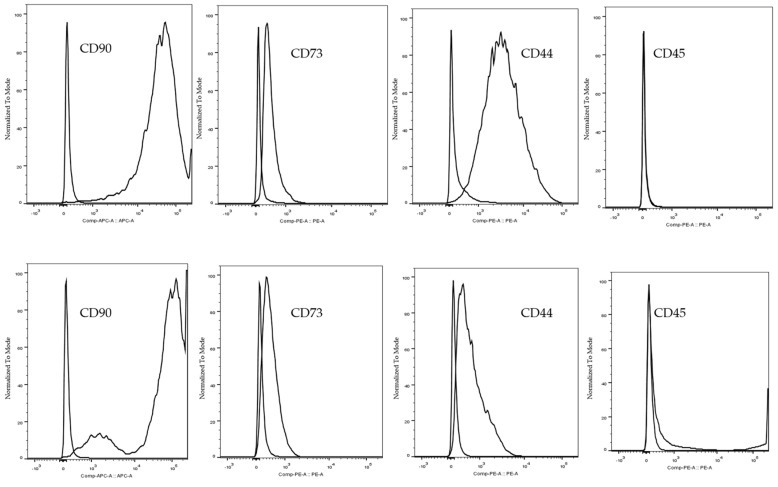
Surface markers of rBMSCs and rDPSCs. Flow cytometry analyses revealed that both cells were positive for CD90, CD73, and CD44 but negative for CD45.

As observed by phase-contrast microscopy, both BMSCs and DPSCs showed a spindle-like fibrous morphology of uniform shape and size (Figure 1). The difference in cell morphology between cells was negligible. Over time, the number of cells increased gradually. The surface antigens of rBMSCs and rDPSCs were analyzed using flow cytometry. FlowJo software (TreeStar Inc., Ashland, OR, USA) was used to analyze the data. As shown in Figure 2, both the cell types were positive for CD90, CD73, and CD44 but negative for CD45.

### 2.2. Multipotent Differentiation of rBMSCs and rDPSCs

**Figure 3:**
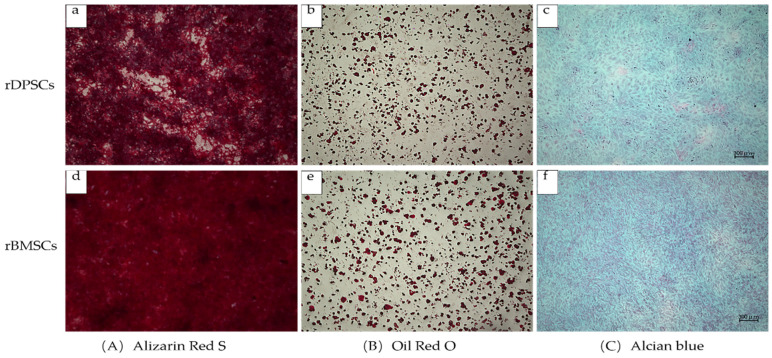
In vitro pluripotency differentiation of cultured rDPSCs (**a**–**c**) and rBMSCs (**d**–**f**). Scale bar = 300 µm. (**A**) Osteogenic differentiation for 21 days followed by staining with Alizarin Red S. (**B**) Adipogenic induction and differentiation for 21 days followed by staining with Oil Red O. (**C**) Induced cartilage differentiation for 21 days followed by staining with Alcian blue.

We evaluated the osteogenic, chondrogenic, and adipogenic differentiation potential of rBMSCs and rDPSCs using different induction media in vitro (Figure 3). In the osteogenic differentiation medium, both cell types showed similar solid mineral deposits. Moreover, under Oil Red O and Alcian Blue staining, both types of cells showed staining for chondrogenic and adipogenic differentiation. No significant differences were found between the chondrogenic and adipogenic differentiation potentials of the two stem cell types.

### 2.3. Production of CellSaic and Scanning Electron Microscope (SEM) Imaging

**Figure 4:**
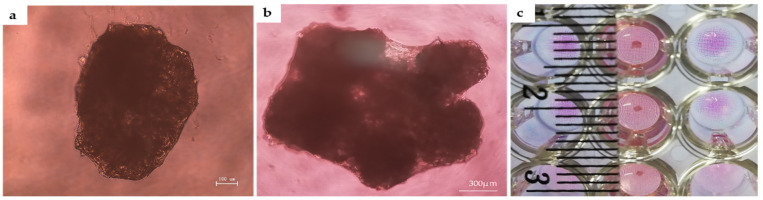
Images of rDPSC-CellSaic under a microscope and after fusion. (**a**) rDPSC-CellSaic image (Scale bar = 100 µm); (**b**) After fusion with eight CellSaics (Scale bar = 300 µm); (**c**) Visual observation of the fused CellSaic.

**Figure 5:**
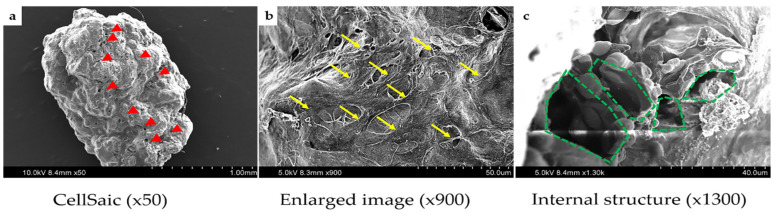
Morphology and structure of rDPSC-CellSaic under a scanning electron microscope. (**a**) Stem cells adhere to each other in rDPSC-CellSaic (the arrows point to the stomata); (**b**) Enlarged image (the arrows point to the cells); (**c**) Internal structure (polygons point to pores).

When the cells were mixed with the scaffold, the cells were in the outer layer of the scaffold. After a day of culture, as shown in Figure 4a (due to the same result for rBMSC, only rDPSC was used as an example for demonstration), the rDPSCs and the scaffold fused to form a rDPSC-CellSaic. The rDPSC-CellSaic samples were placed in the same incubator for 2 days to produce a natural fusion display, which is a way to increase the size of the rDPSC-CellSaic (Figure 4b,c). As long as the support is continuously provided, the size of the rDPSC-CellSaic may further increase, and when other petal-like fragments are not added, the size of the rDPSC-CellSaic does not change [11]. The cell morphology of the rDPSC-CellSaic was observed under a scanning electron microscope (SEM) following 21 days of osteogenic differentiation. There was a significant increase in the number of cells and collagen secretion. The stent structure was stable, and the stomata were visible inside, which is conducive to the exchange of nutrients (Figure 5).

### 2.4. Picro Sirius Red Staining

**Figure 6:**
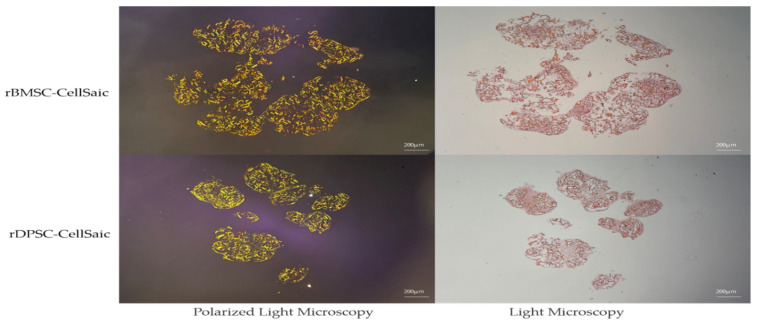
Picro Sirius Red staining under polarized light microscope and ordinary light microscope (Slice thickness = 5 µm, Scale bar = 200 µm).

Eight samples of rBMSC-CellSaic and rDPSC-CellSaic were naturally fused for 2 days, and then stained. Under a polarized light microscope, because the background is black, the scaffold is yellow-orange and/or yellow-green, and the cells are not colored due to a lack of birefringence. Observed by an ordinary optical microscope, the cells adhere to the surface of the scaffold, the wrapped scaffold is orange-red, and the cells are pink (Figure 6).

### 2.5. DAPI and Alizarin Red Staining

**Figure 7:**
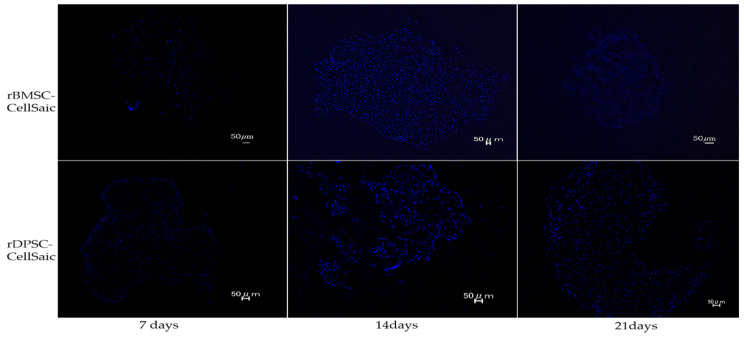
DAPI staining confirmed the presence of cells inside CellSaic after culturing for 7, 14, and 21 days (Slice thickness = 5 µm, Scale bar = 50 µm).

DAPI staining was used to determine the nucleus morphology of cells present inside CellSaic. Strong blue fluorescence was observed under an electron fluorescence microscope, indicating the presence of cell nuclei at 7, 14, and 21 days after culturing (Figure 7).

**Figure 8:**
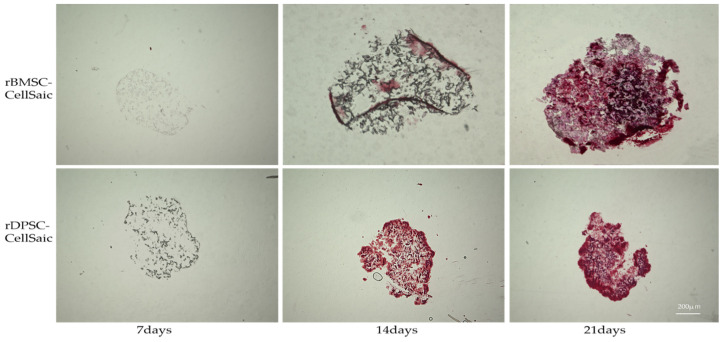
Comparison of the osteogenic differentiation ability of rBMSC-CellSaic and rDPSC-CellSaic in vitro. Alizarin red staining was used to determine osteogenic differentiation after culturing for 7, 14, and 21 days. (Slice thickness = 5 μm, Scale bar = 200 µm).

Alizarin red staining was used to observe the deposition of the osteogenic calcium matrix of rBMSC-CellSaic and rDPSC-CellSaic 7, 14, and 21 days after the induction of culture (Figure 8). After 7 days, no mineral deposits were observed, while the mineral matrix deposition of rDPSC-CellSaic was significantly higher than that of rBMSC-CellSaic after 14 days. After 21 days, no significant differences in red intensity were observed between the two groups.

### 2.6. Real-Time Reverse Transcription Polymerase Chain Reaction (RT-PCR) Measures the mRNA Expression of CellSaic

**Figure 9:**
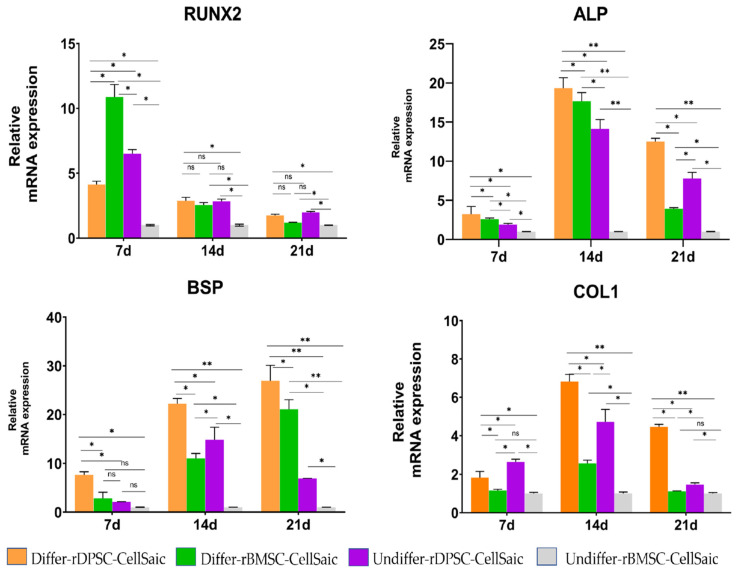
Expression of genes related to bone formation of rBMSC-CellSaic and rDPSC-CellSaic after culturing in normal and osteogenic differentiation media for 7, 14, and 21 days. Differ-rD-CellSaic and Differ-rB-CellSaic were osteogenic differentiation cultures; Undiffer-rD-CellSaic and Undiffer-rB-CellSaic were normal cultures (ns: no significance, * *p* < 0.05, ** *p* < 0.001).

The in vitro osteogenesis of differentiated and undifferentiated rDPSC-CellSaic and rBMSC-CellSaic was determined by the mRNA expression of *RUNX2, ALP, BSP,* and *COL1* (Figure 9). The expression levels of various genes in undifferentiated rBMSC-CellSaic were used as quantitative standards. In the expression of *RUNX2*, both the differentiated and undifferentiated groups of rDPSC-CellSaic and rBMSC-CellSaic showed high expression of *RUNX2* on day 7, which was significantly higher than that on days 14 and 21 (*p* < 0.05). Another mineralization marker, *BSP*, was continuously expressed in the rBMSC-CellSaic differentiation group and rDPSC-CellSaic differentiation group at 7, 14, and 21 days, but there was a decreased expression on day 21 in the rDPSC-CellSaic undifferentiated group; for *COL1* and *ALP*, the expression was significantly upregulated in the differentiated and undifferentiated groups and reached a peak on day 14. The *ALP* expression of the undifferentiated rDPSC-CellSaic group was higher than that of the differentiated rBMSC-CellSaic group on day 21, and the expression of *COL1* in the undifferentiated rDPSC-CellSaic group was always higher than that of the differentiated rBMSC-CellSaic group (*p* < 0.05).

### 2.7. Live/Dead Staining

**Figure 10:**
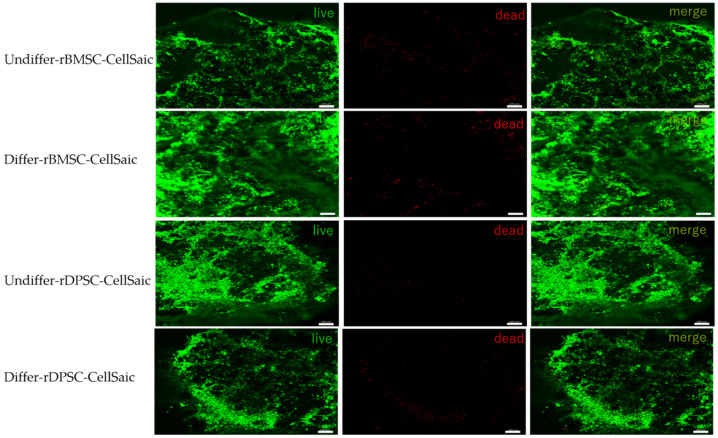
rBMSC-CellSaic and rDPSC-CellSaic were stained for live/dead staining after 21 days of differentiated and undifferentiated culture. Cells stained in green represent live cells, and in red represent dead cells. The cells are fusiform and adhere to each other. (Scale bar = 100 μm).

rBMSC-CellSaic and rDPSC-CellSaic were cultured in differentiated and undifferentiated media for 21 days and then subjected to live/dead staining. A large area of live cell staining can be observed, and there are only a few or no dead cells in the center of CellSaic. It shows that the structure of CellSaic can prevent excessive central accumulation of cells which can lead to death (Figure 10).

### 2.8. Bone Formation and Histological Observations in Rat Congenital Cleft-Jaw Model

**Figure 11:**
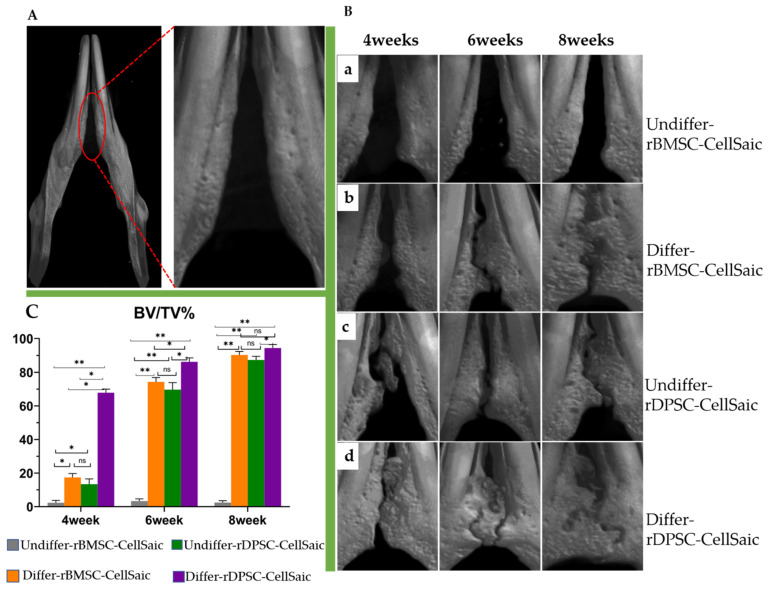
Osteogenesis after 4, 6, and 8 weeks using microcomputed tomography and analysis of bone volume/tissue volume. (**A**) Blank control group 4 weeks after the operation. (**B**) Osteogenesis at 4, 6, and 8 weeks; (**a**) rBMSC-CellSaic undifferentiated group; (**b**) rBMSC-CellSaic bone differentiated group; (**c**) rDPSC-CellSaic undifferentiation group; (**d**) rDPSC-CellSaic differentiation group. (**C**) Analysis of bone volume/tissue volume (%). (* *p* < 0.05, ** *p* < 0.001, ns: no significance).

**Figure 12:**
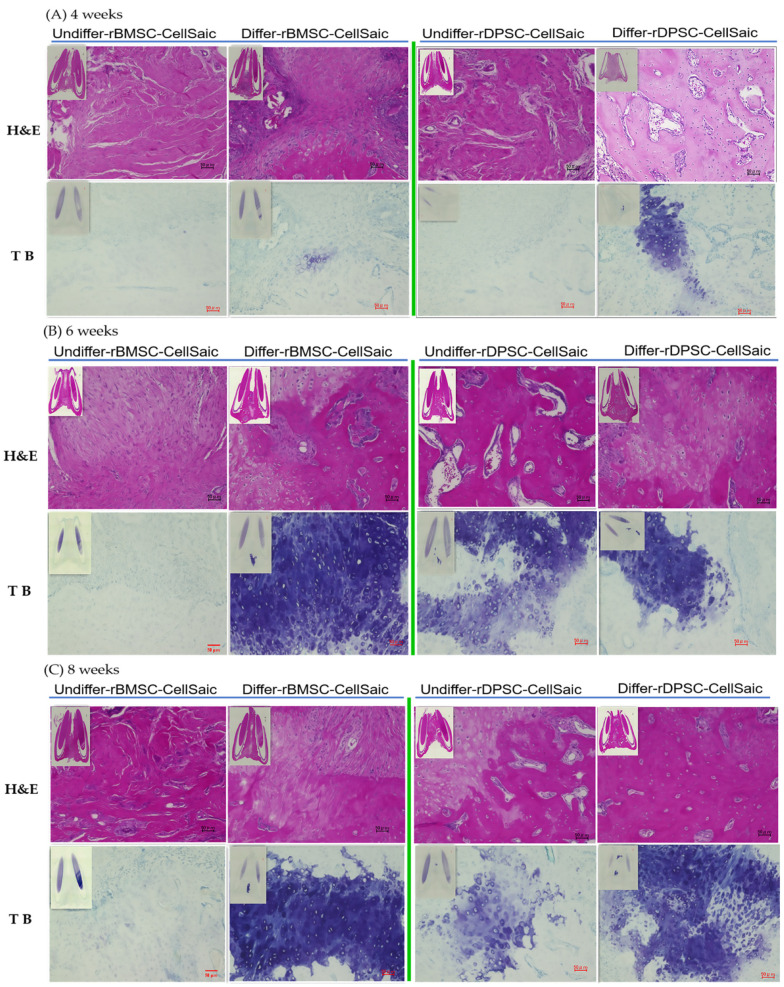
Histological images of bone regeneration at (**A**) 4 weeks, (**B**) 6 weeks, and (**C**) 8 weeks. Sections were stained with hematoxylin–eosin (H&E) and toluidine blue (TB). Upper, small inset image: the image under a low magnification lens; bottom image: high magnification image (scale bar = 50 µm).

First, we fused eight CellSaic to create one cluster and then using four cell clusters, implanted them into a rat model having a congenital cleft palate. After 4, 6, and 8 weeks, tissue samples were collected for bone morphology analysis using microcomputed tomography (μCT) and histological evaluation (Figure 11 and Figure 12). The results showed that the bone volume/tissue volume ratio (BV/TV%) of the defect area in the osteogenic differentiated group was higher than that in the undifferentiated group (Figure 11C). Specifically, in the undifferentiated rBMSC-Cellsaic group, no bone defects or newly formed bone bridges were found at 4, 6 and 8 weeks (Figure 11a). In the differentiated rBMSC-CellSaic and rDPSC-CellSaic osteogenic groups, the 3D reconstructed μCT image of the bone defect showed that the new bone was fused with the host bone 4 weeks after implantation (Figure 11b,d). The undifferentiated rDPSC-CellSaic group had minimal bone formation at week 4 and showed a stronger bone formation from week 6 (Figure 11c).

Upon histomorphological analysis, the data for all experimental groups did not show inflammation or infection due to transplanted materials. At week 4, osteoblasts were observed in the differentiated osteogenic rDPSC-CellSaic and rBMSC-CellSaic culture groups, but no osteoblasts were found in the undifferentiated rDPSC-CellSaic and rBMSC-CellSaic culture groups. However, in weeks 6 and 8, osteoblasts increased in the differentiated osteogenic rDPSC-CellSaic and rBMSC-CellSaic culture groups, while osteoblasts were also found in the undifferentiated rDPSC-CellSaic group. However, in the undifferentiated rBMSC-CellSaic group, only fibrous tissues were observed without osteoblasts. Previous studies reported the formation of cartilage in the jaws of rats when implanted with bone marrow stromal cells with or without β-tricalcium phosphate [23,35]. Consistent with these results, we found that chondrocytes appeared at the junctions of newly formed bones (Figure 12). However, in the absence of bone formation, for example, in undifferentiated rBMSC-CellSaic groups, no chondrocytes were observed at weeks 4, 6, and 8. Table 1 shows a summary of osteogenesis in the congenital cleft palate model.

## 3. Discussion

In previous studies, many experiments have used rat bone defect models, such as those related to skull bones, jawbones, and long bones [14,36,37,38,39], to evaluate the osteogenic ability of biomaterials. Most of these models relied on bone defects produced through surgery, which undoubtedly stimulate the body’s self-repair function, but cannot truly reflect the osteogenic ability of new biomaterials [40]. Recently, Yaguu et al. [41] reported that establishing the rat mandibular union as a congenital split jaw model is of great significance for clinical research. Sasayama et al. demonstrated the feasibility of a rat mandibular congenital defect model for osteogenesis by combining chemically modified gelatin with epigallocatechin gallate and dedifferentiated fat cells [40]. To the best of our knowledge, this study is the first attempt to use a rat mandibular congenital defect model with CellSaic 3D constructs to comprehensively evaluate the osteogenic differentiation ability of rBMSCs and rDPSCs in vivo and in vitro.

In clinical applications, autologous bone transplantation has always been the gold standard [13,42]; however, the combination of stem cells, scaffolds, and osteoinductive agents is considered a prospective method for bone disease repair [43]. Among them, the scaffold plays an important role in stimulating cell proliferation and differentiation and providing growth and nutritional factors for bone defect repair [44]. In this study, the CellSaic 3D construct was specially produced as a cell transplantation platform for MSCs. The cells were cultured in vitro for 21 days, and bone formation still occurred, indicating that the CellSaic platform can help cells to have long-term differentiation activity. Only four weeks after implantation in the body, connective tissue and a small amount of bone tissue appeared in the space of the defect. This result shows that CellSaic can maintain space for tissue regeneration in congenital bone defects, making it a substitute for bone defect repair.

In this study, the rBMSCs and rDPSCs showed a similar fibroblast-like morphology and good proliferation ability. Flow cytometry analysis showed positive expression of CD90, CD73, and CD44, and negative expression of CD45. In the multilineage differentiation experiment, both types of cells can be induced into fat, cartilage, and osteogenic differentiation. Therefore, it can be concluded that both cells exhibit characteristics of multipotent stem cells [45]. We produced CellSaic according to the experimental manual. The diameter of a single CellSaic structure is 0.1–0.5 mm. Due to its good intercellular interaction, it can be fused at will to form a larger cell sphere. The fused CellSaic is visible to the naked eye and can be used with tweezers and easily clamped. This superior transplant operability makes CellSaic a possible transplant material suitable for clinical applications [35,46,47]. SEM results show that the scaffold forms a nutrient and waste exchange channel to prevent the excessive accumulation of cells and to ensure that the internal space can also be supplied with nutrients. Under a polarized light microscope, it can be seen that Sirius red staining stains the Cellnest type I collagen fiber scaffold an orange-yellow, and the cells wrap the scaffold and are closely connected. After osteogenic differentiation for 7, 14, and 21 days, for any frozen section with a thickness of only 5 µm, DAPI staining can indicate the cell distribution, which also showed that CellSaic structures enable cells to grow stably. Alizarin red in vitro calcification staining gradually deepened with the passage of culture time, and rBMSC-CellSaic and rDPSC-CellSaic reached the deepest color after 21 days, indicating that the existence of voids in the structure not only help maintain central cell activity, but also prove to be beneficial to cell calcification [48]. Since both rDPSC-CellSaic and rBMSC-CellSaic showed deep staining at 21 days, we used CellSaic 21 days after in vitro differentiation for our experimental research. Based on the results of the live/dead staining, the cells were in a good living condition after 21 days of in vitro culture, regardless of whether in a differentiated or undifferentiated medium.

At the same time, we noticed that the time-dependent expression of bone-related genes in the rDPSC-CellSaic and rBMSC-CellSaic structures in differentiated and undifferentiated cultures showed interesting results. RUNX2 is the earliest marker gene for bone formation, and it is a sign that osteoblasts are beginning to differentiate. It can activate the transcription and expression of bone sialoprotein (BSP) and Col I genes [49]. In this experiment, rDPSC-CellSaic and rBMSC-CellSaic were cultured in both differentiated and undifferentiated cultures. After maintaining the base for three weeks, the gene expression of the early osteoblast marker RUNX2 could still be observed, confirming that the 3D scaffold environment can indeed promote a longer-lasting expression of RUNX2. The activity of ALP is an indicator to measure the early differentiation of osteoblasts. In this experiment, the activity of ALP increased with time, reaching a peak of expression at 14 days, and then returning back to normal, indicating the transition of cells from proliferation to maturation of the cell matrix. At the same time, the BSP gene level in rDPSC-CellSiac and rBMSC-CellSaic cultured at 7, 14, and 21 days after osteogenic differentiation showed an increasing trend with time, where the BSP gene level at each time point was significantly higher than the respective level in the undifferentiated group. This indicates that the supplemented osteogenic medium can promote the mineralization of hydroxyapatite in vitro and increase the binding of calcium and the formation of calcium nodules. Col I is an extracellular matrix protein that can stimulate the adhesion and differentiation of osteoblasts [50]. Over time, the type I expression of collagen also supports cells that are gradually mineralizing from the collagen matrix, and ALP and Col I showed the same trend, suggesting that there was a cascade reaction between ALP and Col I.

Although undifferentiated multipotent stem cells have the uncertainty of differentiation and are rarely used to directly evaluate the effect of bone regeneration treatment [51], we found an interesting result in this study. In the undifferentiated rBMSC-CellSaic, no new bone formation was found at 4, 6 and 8 weeks, but the undifferentiated rDPSC-CellSaic was able to induce new bone formation in the fourth week. Since the undifferentiated culture of rDPSC-CellSaic is an easy-to-obtain cell source for orthopedics and tissue engineering strategies, this discovery may have value for bone tissue regeneration in the future. On the other hand, at 6 and 8 weeks, both differentiated and undifferentiated rDPSC-CellSaic successfully induced bone formation, which was significantly higher than that in the rBMSC-CellSaic group (*p* < 0.05). Because there is no ethical controversy in obtaining dental pulp stem cells [28,29], it is more feasible to prepare a sufficient number of DPSCs than to obtain BMSCs. It has been reported that the results of combining BMSCs and DPSCs with biological scaffolds to form bone are inconsistent, resulting in an unclear practicability of DPSCs [52,53,54,55]. The results of this study indicate that rDPSC-CellSaic has a better ability to promote bone regeneration than rBMSC-CellSaic. At the same time, after adding osteogenic differentiation culture, the osteogenic ability of the rBMSC-CellSaic and rDPSC-CellSaic groups was significantly higher than that of the undifferentiated control groups, indicating that adding dexamethasone to the culture medium is an effective technique for inducing differentiation of osteogenic MSCs [56,57].

Histological staining showed that there were a large number of osteoblasts in the defect of the congenital mandible, which indicated that rDPSC-CellSaic and rBMSC-CellSaic have good biocompatibility, which is conducive to the attachment of osteoblasts and induces new bone formation. At the same time, toluidine blue staining showed blue-stained chondrocytes, which indicates that the extracellular matrix was rich in mucus polysaccharides, where most of them appeared in the process of bone defect healing. Sasayama et al. previously published research results obtained under similar conditions [40], showing that new osteoblasts were gradually formed from the center to both sides according to the process of cartilage osteogenesis.

In this study, rDPSC-CellSaic may induce the production of new bone in the model, even in the case of an undifferentiated culture. Thus, this study could provide a new feasible option for reducing the cost of clinical applications in the future. At the same time, according to the research results of Marrazzo et al. [58] and Govindasamy et al. [59], we will introduce serum-free culture in follow-up experiments to verify the osteogenic effect, so as to provide richer prospective research results.

## 4. Materials and Methods

### 4.1. Animals and Ethics

The MSCs in this study were obtained from Fisher F344 rats (male, 6-week-old) and 8-week-old Fisher F344 rats were used for the analysis of mandibular defect regeneration. The laboratory animals were kept in the central animal facility of Osaka Dental University to provide a suitable environment. All animal experiments were approved by the local ethics committee of Osaka Dental University and strictly adhered to the guidelines (approval number: 2102004).

### 4.2. Preparation of Dental Pulp Stem Cells (DPSCs) and Bone Marrow Stem Cells (BMSCs)

6-week-old F344 rats were sacrificed by cervical dislocation and were disinfected with 70% alcohol. Approximately 1 mL of bone marrow was harvested from the tibia and femur and suspended in 5 mL α-minimal medium (α-MEM) (FUJIFILM Wako Co., Osaka, Japan), followed by centrifugation at 250 g for 5 min in a 15 mL centrifuge tube at 25 °C. The rat incisor was then removed, and the pulp tissue was carefully obtained. Next, one-third of the root tip was removed, and the tissue was washed three times with phosphate-buffered saline (PBS). This tissue was cut into small pieces, placed in 0.3% type I collagenase, and digested at 37 °C for 1.5 h. It was then centrifuged at 250 g in a 15 mL centrifuge tube for 6 min, and the supernatant was removed.

We added 20% heat-inactivated fetal bovine serum (FBS) and 1% antibiotic antifungal agent (Gibco Invitrogen, Carlsbad, CA, USA) to α-MEM medium as a normal medium. Two types of cells were collected and incubated for 24 h. The medium was changed on the second day to remove nonadherent cells and then changed every 3 days. When the respective cultures reached ≥ 80% confluence, we used 0.5% trypsin-EDTA solution to separate the two kinds of cells and passage them. The cells obtained at passages 2–4 were used for in vitro experiments.

### 4.3. Flow Cytometry Analysis

The characteristic cell surface markers of MSCs were evaluated using flow cytometry. Cells (2 × 10^6^) were obtained after treatment with 0.25% trypsin-EDTA solution (Thermo Fisher Scientific Inc. Waltham, MA, USA). Cell surface antigen staining was performed in PBS containing 2% newborn calf serum (Bovogen Biologicals, Keilor East, VIC, Australia). The cell suspension was incubated with specific antibodies for 30 min at 4 °C. Using FACSVerse (BD Biosciences, Franklin Lakes, NJ, USA) to analyze the cell suspension, we evaluated the following surface antigens with antibodies: CD44 (Cat#12–0444–80, Thermo Fisher Scientific Inc., Waltham, MA, USA), CD45 (Cat#554878, BD Pharmingen, San Diego, CA, USA), CD73 (Cat#bs-4834r-pe, Bioss Antibodies Inc., Boston, MA, USA), and CD90 (Cat#202526, BioLegend, San Diego, CA, USA). The collected data were further analyzed using FlowJo X software (TreeStar Inc., Ashland, OR, USA). The experiment was repeated three times.

### 4.4. In Vitro Functional Multilineage Differentiation

To induce osteogenic differentiation, passage 2 containing 1.5 × 10^5^ cells was placed in a basal medium containing dexamethasone (100 nM/L), L-ascorbic acid (50 µM/L), and β-glycerophosphate (10 mM/L) osteogenic supplement medium for differentiation. The medium was changed every 3 days, and differentiation culture was performed for 21 days. To evaluate the degree of mineralization, the osteogenic cells were washed with PBS, fixed with 4% paraformaldehyde, and then stained with Alizarin Red S solution (Sigma-Aldrich, St. Louis, MO, USA) for 10 min at 25 °C. The mixture was then rinsed five times with distilled water.

To induce adipogenic and chondrogenic differentiation, cells were plated and cultured in adipogenic (MSC-ADDM differentiation medium, Cosmo Bio Co., Ltd., Tokyo, Japan) and chondrogenic (PRIME-XV chondrogenic differentiation medium, Irvine Scientific, Santa Ana, CA, USA) differentiation media for 21 days. The cells were stained with Oil Red O to assess the degree of adipogenic differentiation. After fixing with 4% paraformaldehyde, the cells were washed with 60% isopropanol, stained with Oil Red O solution (Wako, Japan) at room temperature for 10 min, and washed five times with distilled water. Cartilage differentiation was performed by staining the cells with 1% Alcian Blue for 5 min, cells were then washed with distilled water and 250 µL of 3% acetic acid, stained with 0.1% Safranin O (Sigma-Aldrich, St. Louis, MO, USA) for 5 min, and rinsed five times with distilled water. After staining, the cells were observed under an all-in-one fluorescence microscope (BZ-9000; Keyence Co., Osaka, Japan). The experiment was repeated twice.

### 4.5. Production of CellSaic and SEM Images

The CellSaic was prepared by mixing rBMSC (1 × 10^5^ cells/mL) or rDPSC (1 × 10^5^ cells/mL) with Cellnest™ (0.1 mg/mL) (FUJIFILM CO., Minato, Tokyo, Japan) in α-MEM medium. This mixture (200 µL/well) was inoculated on a PrimeSurface 96U plate (Sumitomo Bakelite Co., Ltd., Tokyo, Japan). Each plate was centrifuged using a benchtop plate centrifuge (600 g, 5 min), and then incubated for 24 h. After 21 days of osteogenic differentiation, imaging was performed using a scanning electron microscope at 5 kV or 10 kV acceleration voltage capture (FE-SEM; S-4800; Hitachi; Tokyo, Japan).

### 4.6. Picro Sirius Red Staining

The rBMSC-CellSaic and rDPSC-CellSaic that were naturally fused for 2 days were separated into 5-µm-thick paraffin sections (*n* = 3). The sections were deparaffinized and hydrated with distilled water, covered using the Picro Sirius Red Stain Kit (FUJIFILM Co., Minato, Tokyo, Japan), incubated for 60 min, then washed with acetic acid solution and absolute alcohol, and finally dehydrated, clarified, and mounted. The sections were examined on a microscope (Olympus, Tokyo, Japan) using brightfield or polarized light. Images were collected and analyzed on a camera (Leica DFC 290, Leica Microsystems Ltd., Heerbrugg, Germany) mounted on the vertical tube of the microscope.

### 4.7. Cell Attachment and Calcium Deposition Assay

For cell attachment measurement, the CellSaic was fixed with 4% phosphate paraformaldehyde buffer, and frozen thin sections (thickness 5 µm) were prepared. After washing with PBS, the cells were permeabilized with 0.1% Triton X-100, and DAPI-Fluoromount-G (SouthernBiotech, Birmingham, AL, USA) was used for nuclear staining.

For the determination of calcium deposition, the CellSaic was cultured in osteogenic medium for 7, 14, and 21 days, during which frozen thin sections (thickness of 5 µm) were prepared, and 2% Alizarin Red S solution (Sigma-Aldrich, St. Louis, MO, USA) was used to stain calcium deposits in the extracellular matrix of BMSC-CellSaic and DPSC-CellSaic for 10 min at room temperature, and then washed with PBS. A BZ-9000 digital microscope (Keyence Co., Osaka, Japan) was used to capture all the images. The experiment was repeated three times.

### 4.8. Real-Time PCR (RT-PCR) Analysis

Real-time PCR was used to check the mRNA expression levels of *ALP1*, *COL1A1*, *BSP*, and *RUNX2* in rBMSC-CellSaic and rDMSC-CellSaic. Each sample cultured under each condition was collected at 7, 14, and 21 days after the culture induction. Total ribonucleic acid (RNA) was isolated using the RNeasy Mini Kit (QIAGEN, Hilden, Germany) according to the manufacturer’s protocol. Reverse transcription was performed using the SuperScript^®^ VILOTM cDNA synthesis kit (Invitrogen, Thermo Fisher Scientific Inc., Waltham, MA, USA) with 500 ng RNA. The mRNA levels of the osteogenic markers were partially analyzed using the One Step Plus PCR system (ThermoFisher Scientific Inc., Waltham, MA, USA) using TaqMan gene expression analysis (ThermoFisher Scientific Inc., Waltham, MA, USA). The glyceraldehyde 3-phosphate dehydrogenase (GAPDH) gene was used as an internal standard (rat GAPDH endogenous control; Thermo Fisher Scientific Inc., Waltham, MA, USA). The PCR cycle conditions were as follows: 2 min at 50 °C, 20 s at 95 °C, 1 s at 95 °C, and 20 s at 60 °C, for a total of 40 cycles. Gene expression levels were calculated using the ∆∆CT method. The results are expressed as the fold-change in gene expression level ± standard deviation. The experiment was repeated three times. The accession numbers of the TaqMan gene expression assay PCR system are as follows: COL1A1, Rn01463848_m1; RUNX2, Rn01512298_m1; ALP1, Rn01516028_m1; BSP, Rn00561414_m1; GAPDH, Rn01775763_g1.

### 4.9. Live/Dead Staining

We used the LIVE/DEAD™ Viability/Cytotoxicity Kit for cell detection according to the manufacturer’s instructions (Invitrogen, Thermo Fisher Scientific Inc., Waltham, MA, USA), and all pictures were observed and captured using a Zeiss LSM700 confocal laser microscope (Zeiss, Oberkochen, Germany).

### 4.10. Preparation of the Congenital Cleft-Jaw Model and Sample Implantation

**Figure 13:**
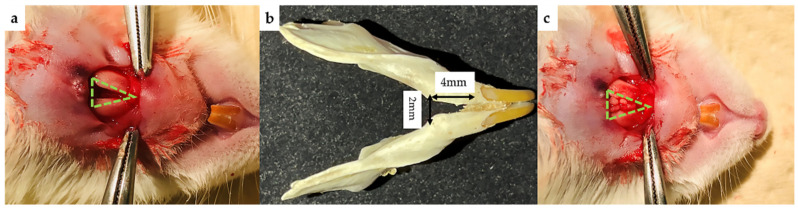
Rat congenital cleft-jaw model. (**a**) Mandibular defect before surgery. (**b**) Size of the mandibular defect model (width 2 mm, height 4 mm, depth 1 mm). (**c**) CellSaic placed into the defect model.

The operation was performed according to a method previously reported by Sasayama [40]. This congenital bone defect in F344 rats (male, 8-week-old) was used to assess osteogenesis (*n* = 3). Preoperative intraperitoneal injection of a medetomidine hydrochloride (0.15 mg/kg; Domitor; Zenoaq, Fukushima, Japan), midazolam (2 mg/kg; Midazolam Sandoz, Sandoz KK, Sandoz KK, Yamagata, Japan), and butorphanol tartrate (2.5 mg/kg; Vetorphale, Meiji Sika Pharmaceutical Co., Ltd., Tokyo, Japan) mixture was used to anesthetize the rats. A skin incision was made in the midline of the mandible, and after muscle dissection, the surgical area was carefully exposed to create a space (Figure 13). The CellSaic, which was cultured 21 days after osteogenic differentiation and 21 days after normal culture as described above, was implanted in each defect, and the skin was covered on the defect and sutured firmly. At 4, 6, and 8 weeks after implantation, mandibular samples were harvested to verify the bone formation ability of each sample. A total of 45 rats were used for the experiment (nine rats × four groups, including nine blank control groups).

### 4.11. Microcomputed Tomography (µCT) Measurement and Histological Analysis

Samples of F344 rats were recovered after 4, 6, and 8 weeks of healing. We used 4% phosphoric acid paraformaldehyde for fixation. To analyze the radiopacity and morphology of the newly formed bone in the defect, a µCT scan (Bruker Skyscan 1172, Bruker, Kontich, Belgium) with a 0.5 mm aluminum filter and 100 mA current under 50 kV radiation was used. The mandible was evaluated, and a 3D image model was then reconstructed using CTAN image analysis software (Bruker, Kontich, Belgium). A cylindrical phantom containing hydroxyapatite (hydroxyapatite content: 200–1550 mg/cm^3^) was used to evaluate the bone mineral density of calcified bone tissue, determine bone volume density (BV/TV%), and express it as an average value ± SD. For histological evaluation, the samples fixed in 4% phosphate paraformaldehyde phosphate buffer solution were decalcified with EDTA, dehydrated, and embedded in paraffin. Thin sections (thickness of 5 µm) were prepared and stained with hematoxylin–eosin and toluidine blue. All images were captured using a BZ-9000 digital microscope (Keyence Corporation, Osaka, Japan).

### 4.12. Statistical Analysis

Statistical analysis was performed using Microsoft Excel software (Microsoft Co., Redmond, WA, USA) and SPSS for Windows (version 25, SPSS Inc., Chicago, IL, USA). All numerical data are expressed as mean ± standard deviation. The differences between the experimental groups were tested using the Tukey–Kramer test or one-way analysis of variance (ANOVA). Differences were considered significant when *p* < 0.05.

## 5. Conclusions

The results obtained in this study indicate that rBMSC-CellSaic and rDPSC-CellSaic made with Cellnest™ as a scaffold, provide excellent support for promoting bone regeneration in rats with congenital mandibular defects. Despite its limitations, this finding suggests that rDPSC-CellSaic can be a better source for craniofacial bone regeneration than rBMSC-CellSaic.

## Figures and Tables

**Table 1:** Summary of bone formation in rat congenital cleft-jaw model (*n* = 3).

	Control	rDPSC-CellSaic		rBMSC-CellSaic	
Implantation Time	Blank Control	Differentiation	Undifferentiation	Differentiation	Undifferentiation
4 weeks	No (3/3)	Yes (3/3)	Yes (1/3)	Yes (3/3)	No (3/3)
6 weeks	No (3/3)	Yes (3/3)	Yes (3/3)	Yes (3/3)	No (2/3)
8 weeks	No (3/3)	Yes (3/3)	Yes (3/3)	Yes (3/3)	No (3/3)

No: no bone formation; Yes: bone formation.

## Data Availability

Data are available upon request.
